# Hazy Transparent Cellulose Nanopaper

**DOI:** 10.1038/srep41590

**Published:** 2017-01-27

**Authors:** Ming-Chun Hsieh, Hirotaka Koga, Katsuaki Suganuma, Masaya Nogi

**Affiliations:** 1 The Institute of Scientific and Industrial Research, Osaka University, 8-1 Mihogaoka, Ibaraki, Osaka 567-0047, Japan

## Abstract

The aim of this study is to clarify light scattering mechanism of hazy transparent cellulose nanopaper. Clear optical transparent nanopaper consists of 3–15 nm wide cellulose nanofibers, which are obtained by the full nanofibrillation of pulp fibers. At the clear transparent nanopaper with 40 μm thickness, their total transmittance are 89.3–91.5% and haze values are 4.9–11.7%. When the pulp fibers are subjected to weak nanofibrillation, hazy transparent nanopapers are obtained. The hazy transparent nanopaper consists of cellulose nanofibers and some microsized cellulose fibers. At the hazy transparent nanopaper with 40 μm thickness, their total transmittance were constant at 88.6–92.1% but their haze value were 27.3–86.7%. Cellulose nanofibers are solid cylinders, whereas the pulp fibers are hollow cylinders. The hollow shape is retained in the microsized cellulose fibers, but they are compressed flat inside the nanopaper. This compressed cavity causes light scattering by the refractive index difference between air and cellulose. As a result, the nanopaper shows a hazy transparent appearance and exhibits a high thermal durability (295–305 °C), and low thermal expansion (8.5–10.6 ppm/K) because of their high density (1.29–1.55 g/cm^3^) and crystallinity (73–80%).

Cellulose nanofibers isolated from plant cell walls have high mechanical strength and modulus, high thermal properties, and are lightweight^1^,^2^,^3^. When films are fabricated with only cellulose nanofibers without any plastics, they exhibit high mechanical properties due to their nanofiber networks and their hydrogen bonding^4^,^5^. These films are termed “nanopapers”. Nanopapers are lightweight and highly foldable, which are similar to conventional paper. However, their appearance is transparent or translucent and not opaque like conventional white paper^6^,^7^,^8^,^9^,^10^,^11^,^12^,^13^,^14^,^15^,^16^,^17^,^18^,^19^,^20^,^21^,^22^,^23^.

When transparent materials scatter incident light, their appearance becomes translucent or opaque. This light scattering is called haze. Materials with low haze are clear transparent, and ones with a high haze are hazy transparent (translucent). When 3–15-nm-wide cellulose nanofibers are densely packed, the nanopaper does not scatter light inside the sheet, and their appearance becomes clear transparent^4^,^5^. Due to their low haze and high thermal properties, clear transparent nanopapers have been developed for optoelectronic device substrates such as transparent electrodes or transistor substrates^6^,^7^,^8^,^9^,^10^. When 3–4-nm wide TEMPO-oxidized cellulose nanofibers were used, the nanopaper had a low haze (less than 1%) and was used as an enhanced gas barrier, which allowed selective permeation of hydrogen gas^11^,^12^.

Some nanopapers have a hazy transparent appearance, which can be applied as high-strength paper and advanced electronic substrates such as organic solar cell and foldable circuits^13^,^14^,^15^,^16^,^17^,^18^,^19^. Frosted glasses are hazy despite their transparent body, because their rough surface scatters incident light. However, hazy transparent nanopaper does not scatter light at their surface, because their surface is as smooth as transparent glass or plastic. Therefore, their light scattering occurs from inside the nanopaper. Previous studies proposed that the light scattering is increased by wider cellulose nanofibers^20^,^21^ or by lower density nanopapers^9^,^22^,^23^. However, the scattering mechanism is still unclear.

In this study, the reasons for scattered transmitting light in hazy transparent nanopaper are discussed. There are many cellulose nanofibers made using mechanically or chemically nanofibrillations. Among them, nanopaper produced from mechanically nanofibrillated cellulose nanofibers are the most promising because of their higher thermal resistance^8^. Moreover, this nanopaper shows various appearance as clear transparent, hazy transparent, and opaque. Thus, using mechanically nanofibrillated nanopaper, their light scattering is presented, and a fabrication method and application of hazy transparent nanopaper is developed.

## Experimental

### Cellulose pulps

Five cellulose pulps were used: a never-dried holocellulose pulp with alkali treatment from softwood (Japanese cedar) or hardwood (eucalyptus) chips, a never-dried softwood or hardwood bleached sulfite pulp, and dried cellulose pulp (KC FLOCK W100-GK). The bleached sulfite pulps and dried cellulose pulps were gifts from Nippon Paper Group. Inc. These pulps had α-cellulose contents over 90% and Klason lignin of 0%. The procedure for the alkali treatment of holocellulose pulp is as follows^8^. Wood chips (50 g) were delignified by heating in an acetic anhydride/hydrogen peroxide mixture (1,000 mL: 1,000 mL) at 90 °C for 4 h. The holocellulose pulp was treated with 5 wt% potassium hydroxide at 20 °C for overnight, and then at 80 °C for 2 h. The alkali-treated holocellulose pulps had α-cellulose contents of 70–85% and Klason lignin of 0%.

### Nanofibrillation

All of the cellulose pulps were fibrillated using a water-jet nanofibrillation system^5^,^8^. In this system, a 0.5 wt% pulp water dispersion of 2,000 g was homogenized using a high-pressure water-jet system (Star Burst, HJP-25008, Sugino Machine Co., Ltd.) equipped with a ball-collision chamber. The dispersion was ejected from a small nozzle with a diameter of 0.17 mm under a high pressure of 245 MPa. The water dispersions were passed through this nozzle up to 100 times. After mechanical nanofibrillation, additional treatments such as centrifugation and filtering are often applied to remove unfibrillated or aggregated fibers. In this study, however, obtained dispersions were directly used for the starting materials of nanopaper without any post treatment.

### Nanopaper

The 40-μm-thick nanopapers were prepared as follows. The nanofiber dispersions (0.4 wt%, 60 g) were vacuum filtered with a glass filter (KG-90, Toyo Roshi Kaisha, Ltd.) and using a mixed cellulose ester membrane filter (A020A090C, Toyo Roshi Kaisha, Ltd. pore size: 0.2 μm). The wet nanofiber sheets were dried at 110 °C for 10 min under an applied pressure of 0.01 MPa. The thickness of nanopaper was measured by a micrometer.

### Characterizations

The cellulose nanofiber/water dispersion was adjusted to 0.1 wt%. The dispersions do not contain any sediments after standing for 1 h. The transmittance of the dispersions were measured at a wavelength of 550 nm using a UV-visible spectrometer (U-3900, Hitachi High-Technologies Corp.) with distilled water as a reference.

Total transmittance and haze of the nanopapers were measured under a D65 light source (HZ-V3, Suga Test Instruments Co., Ltd.). X-Ray diffraction patterns were recorded using a Rigaku MiniFlex600 with Cu-Kα radiation and a scanning angle (2θ) range of 10–30°, at 40 kV voltage and 15 mA current. The crystallinity index of cellulose I was calculated from the (200) reflection (2θ = ca. 22.6°) following the procedure from previous reports^24^,^25^. The thermal durability of the nanopaper was evaluated using the 5% weight loss point. The coefficient of thermal expansion (CTE) was measured using a thermomechanical analyzer (TMA/SS7100, SII Nanotechnology Inc.). The CTE values were determined as the mean values at 20–150 °C in the second run. Thermogravimetric analysis was measured under a nitrogen atmosphere (60 mL/min) at a heating rate of 10 °C/min (TGA Q50N2, TA Instruments).

The nanofiber papers were observed using a field emission scanning electron microscopy (FE-SEM) (SU8020, Hitachi High Technologies Corp.) at the 2,000 or 30,000 times magnification. Before the FE-SEM observation, the samples were platinum coated in an ion sputter coater (Mild Sputter E-1045, Hitachi High Technologies Corp.). For the FE-SEM observation, an accelerating voltage of 1.5 kV and a working distance of 7–8 mm were used.

## Results and Discussion

**Figure 1:**
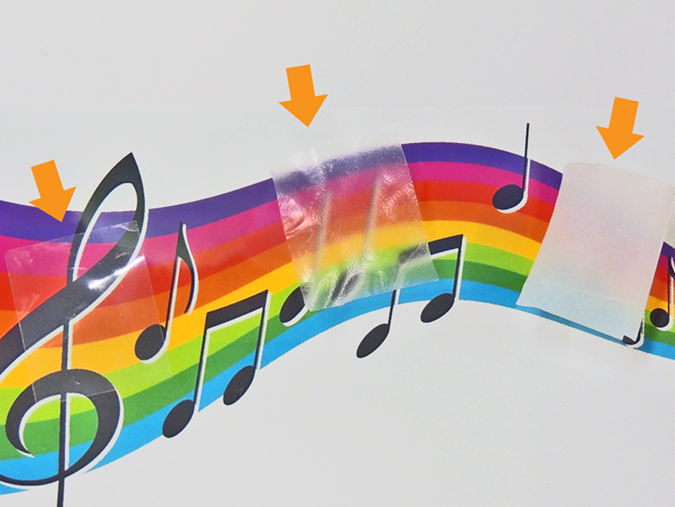
Various appearances of cellulose nanopaper consisting of 15-nm-wide nanofibers. Left: Clear transparent nanopaper with a density of 1.48 g/cm^3^. Center: Translucent nanopaper with a density of 1.15 g/cm^3^. Right: Opaque nanopaper with a density of 0.81 g/cm^3^.

The cellulose nanopaper was fabricated by drying the cellulose nanofiber/water dispersions. When the water dispersion with 15-nm-wide cellulose nanofibers was dried, the cellulose nanofibers densely packed together, and air voids inside the sheets were removed^4^,^5^. As a result, the high-density cellulose nanopaper (1.48 g/cm^3^) became clear transparent with a small haze of 4.2% (Fig. 1 left). Because native cellulose nanofibers are hydrophilic, they do not homogeneously disperse in organic solvents such as ethanol, methanol, and acetone^26^. Therefore, after drying the nanofiber/organic solvent suspensions, numerous air voids remain inside the sheet. Because the air voids cause light to scatter, the nanopaper become translucent or white appearance. For example, when the cellulose nanofiber suspension with the mixture of 50% ethanol/50% water were dried, the density of cellulose was 1.15 g/cm^3^, and the nanopaper was translucent (haze: 49.3%) (Fig. 1 center). Increasing the ethanol to 90% decreased the density to 0.81 g/cm^3^, and the appearance was white translucent (haze: 90.1%) (Fig. 1 right). However, all the samples maintained high total transmittances of 90.3–90.7% because the incident light was transmitted with or without light scattering. Although the samples were fabricated with the same width of cellulose nanofibers, the density of cellulose nanopaper changed their appearance from clear transparent to hazy transparent, which has been reported in previous studies^9^,^22^,^23^. However, the low-density nanopapers show low mechanical properties because these characteristics are related to highly dense cellulose nanofibers. Thus, we propose a method of customizing nanopaper haze to maintain their high density.

**Figure 2:**
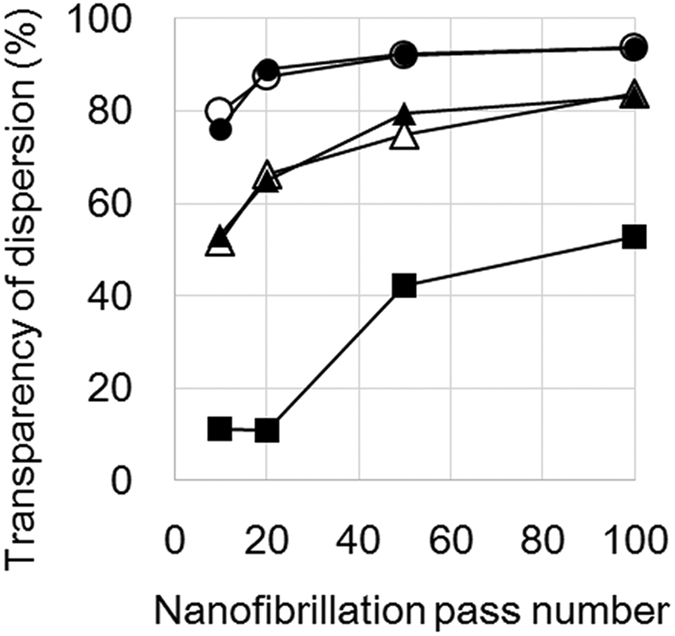
Transparency of a 0.1 wt% cellulose nanofiber dispersion. ○: Low purity cellulose wet pulp from softwood, ●: low purity cellulose wet pulp from hardwood, △: high purity cellulose wet pulp from softwood, ▲: high purity cellulose wet pulp from hardwood, ■: high purity cellulose dried pulp from hardwood.

We prepared three cellulose pulps of “low purity cellulose wet pulp from softwood or hardwood”, “high purity cellulose wet pulp from softwood or hardwood”, and “high purity cellulose dried pulp from hardwood”. Low purity cellulose wet pulps were never-dried wood fibers after removal of all the lignin and most of the hemicellulose. The pulps were suitable to provide 15–nm-wide cellulose nanofibers using mechanical nanofibrillation^27^,^28^. The transmittance of cellulose nanofiber dispersions reached 90% after 20 nanofibrillation cycles (Fig. 2 circles). After removal of all the lignin and almost all of the hemicellulose, the high purity cellulose wet pulps were never-dried and high purity cellulose dried pulps were dried. In these pulps, the cellulose nanofibers were coalesced by irreversible hydrogen bonding from hornification^28^. Thus, despite repeated (100 times) mechanical nanofibrillation, the dispersions transmittances were around 80% for high purity cellulose wet pulps (Fig. 2 triangle) and less than 60% for high purity cellulose dried pulps (Fig. 2 square). Nanofiber dispersions from softwood and hardwood showed the same transmittance under the same conditions of pulp purification or drying (Fig. 2 open and filled circles, open and filled triangles). Nanofiber dispersions with a wide range of transparencies were obtained from various pulp sources by repeated mechanical nanofibrillations.

**Figure 3:**
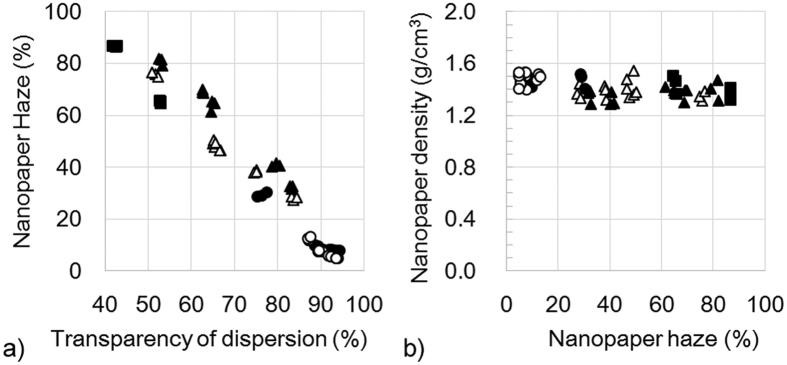
(**a**) Increasing nanopaper haze decreases with transparency for 0.1 wt% cellulose nanofiber dispersions. (**b**) Density of nanopaper is constant against nanopaper haze. ○: Low purity cellulose wet pulp from softwood, ●: low purity cellulose wet pulp from hardwood, △: high purity cellulose wet pulp from softwood, ▲: high purity cellulose wet pulp from hardwood, ■: high purity cellulose dried pulp from hardwood.

When these dispersions were vacuum filtered and dried, 40-μm-thick nanopapers were obtained. The haze value of nanopaper shows a liner relationship with the dispersion transparency (Fig. 3a). A large haze nanopaper was fabricated from a turbid dispersion using pulp from softwood or hardwood. As mentioned above, nanopapers from ethanol dispersions increased their hazes from 4.1% to 90.1% with decreasing densities from 1.48 to 0.81 g/cm^3^. However, these nanopapers show hazes that increased from 4.9% to 86.7%, while maintaining a high density of 1.29–1.55 g/cm^3^ (Fig. 3b). In these hazy transparent nanopaper, their density were less effective to their haze value.

**Figure 4:**
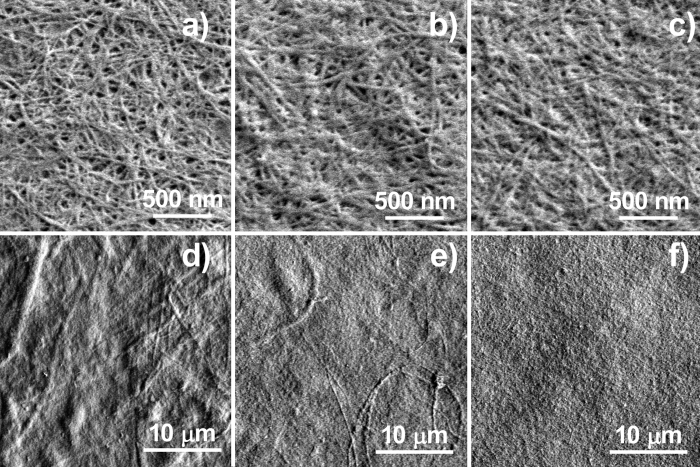
FE-SEM images of cellulose nanopaper of various hazes. (**a**,**d**) 86.6% Haze nanopaper from high purity cellulose dried pulp from hardwood, (**b**,**e**) 38.3% haze nanopaper from high purity cellulose wet pulp from softwood, and (**c**,**f**) 5.5% haze nanopaper from low purity cellulose wet pulp from softwood.

Nanopaper haze was previously reported to increase from 20% to 49% when the nanofiber width was increased from 10 to 50 nm, because of the light scattering at the cross-section of cellulose nanofibers^20^. Therefore, cellulose nanofibers in 86.6%, 38.3% and 5.5% haze nanopapers were observed using FE-SEM at the 30,000 times magnification. However, no nanofibers wider than 50 nm were observed in the hazy transparent nanopaper, and uniform width nanofibers around 15 nm were present in all the nanopapers (Fig. 4a–c). Starting pulp fibers are microsized hollow cylindrical shapes, which are composed of 15–nm-wide cellulose nanofibers. Therefore, when any cellulosic fiber materials were observed at high magnifications, the FE-SEM images show only homogeneous wide cellulose nanofibers around 15 nm. In our previous studies, we propose that light scattering inside the nanopaper was caused by the difference of refractive index between cellulose nanofiber and the air cavities^4^,^5^. The cavity size and their number in these nanopapers were carefully observed, and they were the same size (around 50 nm) and distribution in the hazy and clear transparent nanopapers (Fig. 4a–c). Thus, the nano-order observations did not present any evidence of increasing light scattering in hazy transparent nanopapers.

**Figure 5:**
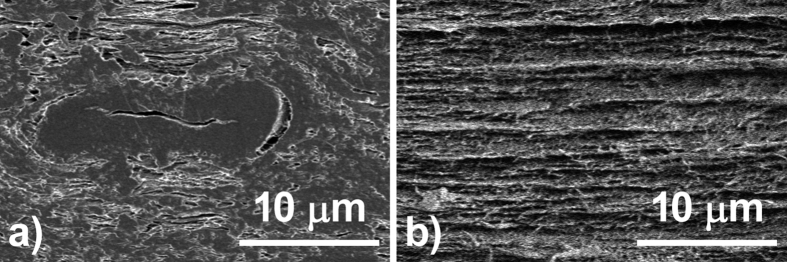
Cross-sectional FE-SEM images of cellulose nanopaper. (**a**) A microsized pulp fiber showing compressed hollow cylinder in the hazy transparent nanopaper (Haze 86.6%), (**b**) Densely laminated layers in the clear transparent nanopaper (haze 5.5%).

Uetani *et al*. reported that pulp fibers were broken into microsized fragments, and the 15-nm-wide nanofibers peeled away from the fragments during mechanical nanofibrillation^29^. To observe microsized fragments in the nanopaper, lower magnifications of 2,000 times were used (Fig. 4d–f). In the clear transparent nanopaper, fragments were not observed from the top, and densely laminated layers were observed from the side (Figs 4f and 5b). In the hazy transparent nanopaper, microsized fragments were observed, and they increased with increasing nanopaper haze (Fig. 4d,e). From the cross-sectional observations, the microsized fibers were compressed, and the cylindrical hollow shape became flat (Fig. 5a). These results show that hazy transparent nanopaper causes light scattering inside the film, and they can maintain their high density as follows. Hazy transparent nanopapers consist of mainly cellulose nanofibers and some microsized cellulose fibers. The microsized fibers were hollow cylinders, but they are compressed and flattened in the nanopaper. The flattened cavities cause light scattering inside the nanopaper, without decreasing the paper density. As a result, the nanopaper is hazy transparent and of a high density.

**Figure 6:**
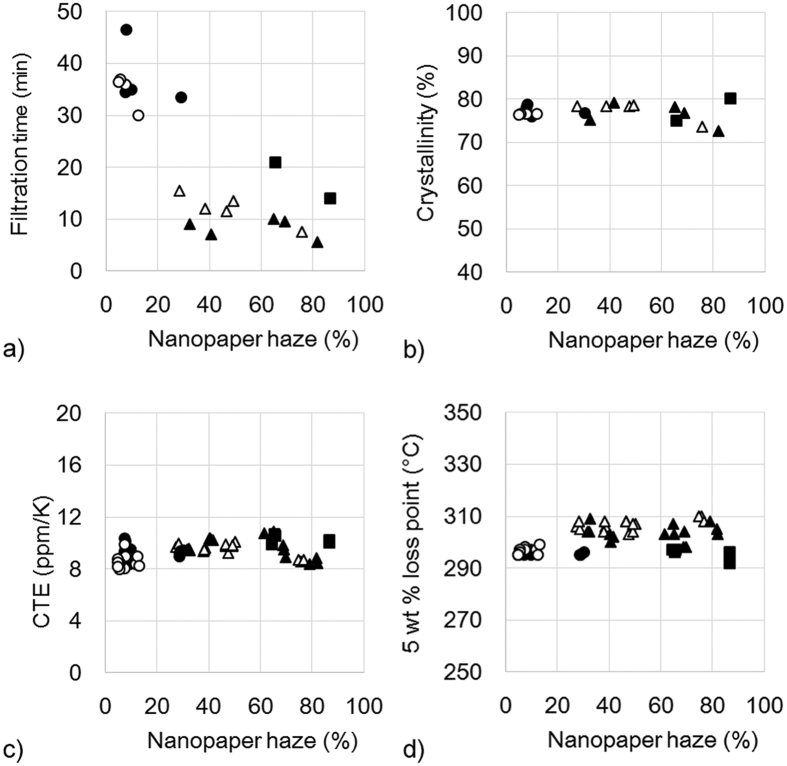
Hazy transparent nanopaper decreases filtration time with increasing haze (**a**), maintains a high crystallinity (**b**), shows low CTEs (**c**), and exhibits a high heat resistance under nitrogen (**d**). ○: Low purity cellulose wet pulp from softwood, ●: low purity cellulose wet pulp from hardwood, △: high purity cellulose wet pulp from softwood, ▲: high purity cellulose wet pulp from hardwood, ■: high purity cellulose dried pulp from hardwood.

The hazy transparent nanopaper shows another advantage of short fabrication time. Nanopapers were fabricated by vacuum filtering the nanofiber/water dispersion, and the wet sheet was hot-pressed for 10 min. Clear transparent nanopaper was fabricated using the dispersion containing 15-nm-wide cellulose nanofibers. The nanofiber dispersion was a low concentration (0.4 wt%), but of a high viscosity. Thus, the filtration step required over 30 min (Fig. 6a). However, the starting dispersion of hazy transparent nanopaper was not highly viscous because the mixture contained microsized fibers and 15-nm-wide nanofibers. Therefore, their filtering time decreased by one-third to 10 min (Fig. 6a). The hazy transparent nanopaper shows a high density at 1.29–1.55 g/cm^3^ (Fig. 3b) and high crystallinity at 73–80% (Fig. 6b). The CTE was low at 8.5–10.6 ppm/K (Fig. 6c), and the thermal stability was high at 295–305 °C (Fig. 6d), which were similar to the clear transparent nanopaper.

**Figure 7:**
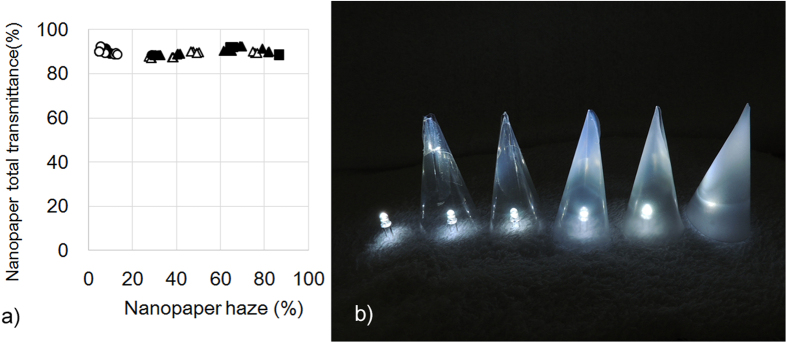
Total light transmittance and haze value of nanopaper (**a**), and the application of hazy transparent nanopaper as optical diffusers (**b**). Far left: an LED light without diffuser, from second-to-left to right: increasing nanopaper haze, which homogeneously spreads the light through the nanopaper and maintains light intensity.

A new application of hazy transparent nanopaper is presented. The hazy transparent nanopaper shows a short fabrication time, high thermal resistance, and low CTE. Furthermore, the hazy transparent nanopaper transmits around 90% incident light (Fig. 7a). These characteristics are a good match for optical diffusors in light emitting diode (LED) lighting. When LEDs were covered with the hazy transparent nanopapers, the nanopaper homogeneously spread the dazzling LED light without losing high light intensity (Fig. 7b). LED lighting has become widespread rapidly, because they have advantages over incandescent light sources including lower energy consumption, longer lifetime, and smaller size. In the near future, ecofriendly lighting with low energy consumptions will be realized using LEDs and hazy cellulose nanopaper.

## Conclusions

There are two transparent nanopapers, one is clear transparent with low light scattering, and the second is hazy transparent with higher light scattering. Hazy transparent nanopaper is produced by reducing of the density of fibers. In this study, a hazy transparent nanopaper with a high density is fabricated using various wood pulps from softwood or hardwood in a dry or wet state. When 15–50-μm-wide cellulose pulp fibers were mechanical nanofibrillated, most fibers became 4–15 nm wide, but some fibers became microsized fragments. These microsized fragments show a hollow cylindrical shape that becomes flat in the nanopaper. The nanopaper shows high density, low CTE, and high thermal durability similar to that of the clear transparent nanopaper. The haze value of nanopaper increases with the number of microsized fragments because the compressed hollow cylinders cause light scattering inside the nanopaper. The hazy transparent nanopaper scatters the incident light without losing light intensity. Therefore, the hazy transparent nanopaper is a promising candidate for optical diffusors in LED lighting.

## Additional Information

**How to cite this article**: Hsieh, M.-C. *et al*. Hazy Transparent Cellulose Nanopaper. *Sci. Rep.* **7**, 41590; doi: 10.1038/srep41590 (2017).

**Publisher's note:** Springer Nature remains neutral with regard to jurisdictional claims in published maps and institutional affiliations.
